# Cytosolic Genomic DNA functions as a Natural Antisense

**DOI:** 10.1038/s41598-018-26487-1

**Published:** 2018-06-04

**Authors:** Ken Asada, Keiya Ito, Daishi Yui, Hirokuni Tagaya, Takanori Yokota

**Affiliations:** 1 Department of Neurology and Neurological Sciences, Graduate School of Medical and Dental Sciences, Tokyo Medical and Dental University, Tokyo, 113-8519 Japan; 2 Center for Brain Integration Research, Tokyo Medical and Dental University, Tokyo, 113-8519 Japan; 3 Department of Health Science, School of Allied Health Sciences, Kitasato University, Sagamihara, 252-0373 Japan

## Abstract

Stress conditions such as UV irradiation, exposure to genotoxic agents, stalled DNA replication, and even tumors trigger the release of cytosolic genomic DNA (cgDNA). Classically, cgDNA induces interferon response via its binding to proteins such as STING. In this study, we found previously reported cgDNA (cg721) exists in the cytosol of the mouse cell lines, cultured under no stress conditions. The overexpression of cg721 suppressed the complementary RNA expression using strand selection and knockdown of DNA/RNA hybrid R-loop removing enzyme RNase H and three prime repair exonuclease 1 TREX1 increased the expression levels of cg721 and thus, inhibited the target Naa40 transcript, as well as protein expression, with a phenotypic effect. In addition, cgDNA was incorporated into extracellular vesicles (EVs), and the EV-derived cg721 inhibited gene expression of the acceptor cells. Thus, our findings suggest that cg721 functions as a natural antisense DNA and play a role in cell-to-cell gene regulation once it secreted outside the cell as EVs.

## Introduction

Cytosolic nucleic acids and the sensor proteins they are bound to are being increasingly reported these days (Table S1). Cytosolic nucleic acids can be classified by their origin. One is a pathogenic DNA or RNA invaded from outside, which is recognized by sensor proteins, resulting in the induction of inflammatory pathways and the risk of cancer^1–3^. The other is from within the cell, nuclei. Stress conditions for DNA, including UV irradiation, exposure to genotoxic agents, stalled DNA replication (R-loops formation), and tumors, trigger the release of cytosolic genomic DNA (cgDNA)^4–7^ and cytosolic chromatin^8–10^. Previous two studies demonstrated that (1) 129 cytosolic DNA clones were identified from heart tissue isolated from IRF3 KO mice, while 137 cgDNA clones from TREX1/IRF3 double KO mice^11^ and (2) a total of 231 cytosolic DNA were cloned from tumor cells present in Eμ-Myc mice that is a model of Myc driven malignancy and genotoxic reagent Ara-C treated BC2 cells, a B cell lymphoma cell line derived from Eμ-Myc mice. The length of cytosolic DNA predominantly ranged 100–1,000 for double-stranded DNA (dsDNA) and less than 100 for single-stranded DNA (ssDNA). Most of the cytosolic DNA sequences were matched to endogenous host mouse genome and derived from intergenic and intragenic regions, indicating that they were cgDNAs^7^. The cgDNA found in lymphomas, cancer cells, and mouse embryonic fibroblasts (MEF) has been shown to be associated with the immune response^7,11–13^. dsDNA with any sequences longer than 24 bp induces inflammatory cytokines, IFN-α/β^14^. Signal interfering DNA, a class of short (8–64 bp in length) modified double-stranded DNA molecules, is capable of inhibiting DNA repair activities. dsDNA mimicking double-strand DNA breaks (DSB) activate PARP and cytosolic DNA sensor DNA-PK, while those mimicking single-strand breaks only activate PARP^15,16^.

RNA interference (RNAi) were first found to suppress the target genes in *C. elegans*^17^. Double-stranded RNA (dsRNA) is processed into small interfering RNA (siRNA) by Dicer and siRNA is loaded into the RNA-induced silencing complex (RISC). Endogenously generated small RNAs (miRNA and piRNA), and their degradation mechanisms play an important role in cell development, differentiation, proliferation, metabolic control, transposon silencing, antiviral defense, and tumorigenesis^18–20^. Exogenous application of small and long DNA has been implicated in the inhibition of Tobacco mosaic virus infection in plants^21^. The antisense oligonucleotide of synthesized single-stranded DNA (ASO) suppresses target genes and a beneficial tool for clinical and research purpose. Recently, we have developed ASO and its complementary RNA, DNA/RNA heteroduplex oligonucleotides (HDO). HDO unwinds in the cytosol and the generated parental ASO hybridizes with the target RNA through the sequence specific recognition and cleaves the target RNA by RNase H. We have demonstrated that HDO increased the target gene silencing efficacy compared to the parental ASO, advancing the possibility of oligonucleotide-based therapeutics^22^.

Argonaute (Ago) is a key component of RISC for siRNA and miRNA dependent gene silencing. DNA-guided DNA interference by Ago has been studied previously^23,24^. Prokaryotic Ago can use DNA as a guide strand^25,26^, while eukaryotic Ago uses RNA as a guide. Despite no direct cleavage being observed, ASO interacted with the Ago2-PAZ domain and localized into GW-182 mRNA-degrading bodies^27^. Although cytosolic DNA is thought to be recognized by its sensors to induce immune responses, RNA polymerase III sensitive cytosolic DNA/RNA hybrids bind to the miRNA components and regulate miRNA expression^28^, thereby implying that cgDNA may have additional functions. However, none of the studies, so far, certified cgDNA as a natural antisense to regulate mRNA expression. In this study, we found that knocking down of DNA/RNA hybrid ribonuclease RNase H1 and exodeoxyribonuclease TREX1 increased the expression levels of cgDNA and inhibited the target Naa40 transcript as well as protein expression, with a phenotypic effect.

## Results

### Identification of cytosolic genomic DNA

**Figure 1:**
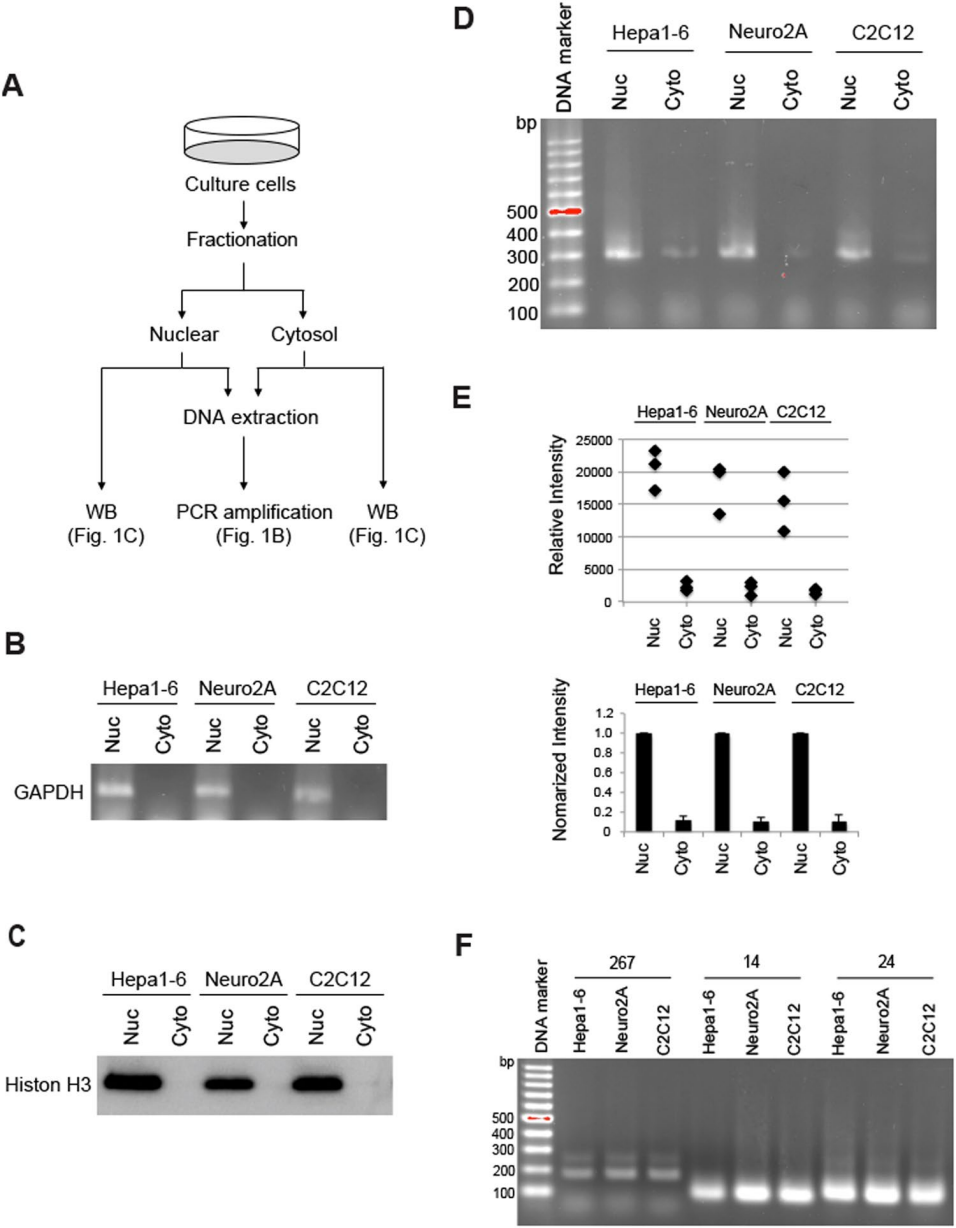
The cytosolic fraction does not contain nuclear components. (**A**) The experimental scheme of fractionation. (**B**) Fractions from three mouse cell lines were used for cgDNA PCR amplification with mouse *Gapdh* intron specific forward and reverse primers. (**C**) Immunoblotting of nuclear and cytosolic fractions. (**D**) Sequences of 721 in nucleus and cytosols from Hepa1-6, Neuro2A, and C2C12 cells were amplified by PCR. (**E**) Semi-quantitative analysis of cgDNA. Images were analyzed by using ImageJ software. The upper panel represents a dot plot. The lower panel represents a normalized bar graph. (**F**) Cytosolic genomic transposon DNA was amplified by PCR.

Nuclear, mitochondrial, and cytosolic fractions were prepared by centrifugation from mouse hepatocellular carcinoma Hepa1-6 cells, mouse brain neuroblastoma Neuro2A cells, and immortalized mouse myoblast C2C12 cells. Prior to conducting the cgDNA study, we first examined whether any nuclear fraction contaminant was present in the cytosolic fraction following the fractionation procedures. RNA extracted from the prepared fractions was amplified by using mouse *GAPDH* primers targeting the intron 4 region to detect nuclear mRNA (pre-mRNA). *GAPDH* was amplified from the nuclear fraction samples (Fig. 1B; Nuc), but not from the cytosolic fraction samples^7^ (Fig. 1B; Cyto). Moreover, western blotting showed that nuclear protein Histone H3 was detected only in the nuclear fractions (Fig. 1C; Nuc) but not in the cytosolic fractions (Fig. 1C; Cyto), indicating that no nuclear components were carried over to the cytosolic fractions. In addition, we analyzed mitochondrial contaminants by western blotting of VDAC1, an outer mitochondrial membrane (Fig. S1A). VDAC1 was only detected in the mitochondria fractions (Fig. S1A; Mito), but not in the cytosolic fractions (Fig. S1A; Cyto). This observation suggested that neither nuclear nor mitochondria components existed in the cytosolic fractions.

Although PCR is known as a qualitative rather than a quantitative method, we normalized the DNA template to 30 ng per reaction in all experiments and, thus, PCR assays were regarded as semi-quantitative in our study. We first analyzed the expression of a previously reported cgDNA^7^ (referred to as cg721, 286 bp long) under physiologically normal conditions. As shown in Fig. 1D, cg721 was detectable without any toxic reagent treatment in both tumorous Hepa1-6 and Neuro2A cells and in non-tumorous C2C12 cells. Figure 1E top shows the relative intensities from Fig. 1D and bottom represents a normalized bar graph for each nuclear intensity. The cytosolic fractions yielded 6 to 11% of the amplified products compared to the nuclear fractions in all three cell lines (Fig. 1E bottom and Fig. S1B). To address whether cgDNA occurrence is a general phenomenon, we tested other known cgDNAs of transposon origin, which are robustly increased in TREX1 knockout MEF cells^11^ (hereafter, clone name WT-267 is referred to as 267 (160 bp long), WT S-24 as 24 (98 bp long), and WT S-14 as 14 (82 bp long)). The 267, 24, and 14 were all detectable, indicating that not only cg721 but also transposon DNAs were present in the cytosol (Fig. 1F). Gasser and colleagues reported that cgDNA was present in B-cell lymphoma cell lines such as BC2, treated with the genotoxic replication inhibitor Ara-C^29^ or untreated Eμ-*Myc*, Yac-1, or human lung carcinoma cell line, A549^7^. Shibata and Dutta identified many of short circular DNAs in mouse tissues, as well as mouse and human cell lines^30^. Here, we found that cgDNA exists, under physiological conditions, at levels sufficient for PCR amplification as seen in two tumorous and one non-tumorous mouse cell lines.

### cgDNA triggers DNA interference

**Figure 2:**
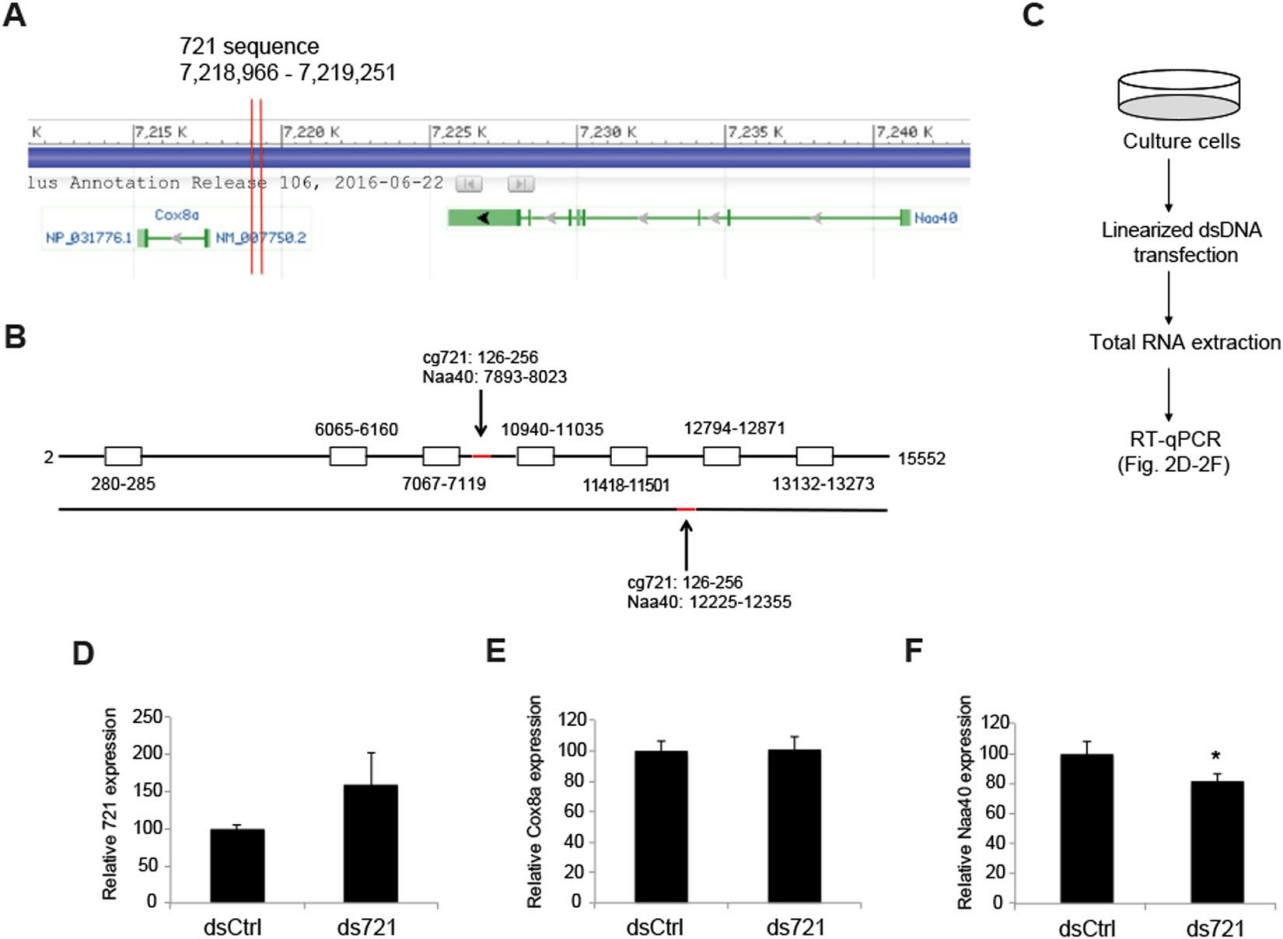
Gene mapping of 721 sequence on chromosome 19. (**A**) 721 sequence located on the upstream of Cytochrome c oxidase subunit VIIIa (Cox8a) and the downstream of N(alpha)-acetyltransferase 40, NatD catalytic subunit (Naa40). (**B**) The sense strand of the 721 sequence mapped to both the sense and antisense strands of Naa40. (**C**) The experimental scheme of RT-qPCR. (**D**–**F**) Ctrl or 721 dsDNA 4 nM were transfected to C2C12 mouse muscle cells. RNA was extracted 24 h post-transfection. Quantitative analysis of the liDNA effect for (**D**) 721, (**E**) Cox8a and (**F**) Naa40 (p < 0.05; n = 3; mean ± SD).

We next conducted bioinformatics analysis to determine whether cg721 maps to other genomic regions. cg721 locates to genomic region 7,218,465 to 7,219,750 on chromosome 19,849 bp upstream of the gene encoding Cytochrome c oxidase subunit VIIIa (Cox8a) and 5,918 bp downstream of the gene encoding N(alpha)-acetyltransferase 40, NatD catalytic subunit (Naa40) (Fig. 2A). The non-coding region of 721 was transcribed from the genome (Fig. S1C; left three lanes), indicating that it may overlap with the 5′-UTR of Cox8a and/or 3′-UTR of Naa40. Furthermore, with interest, cg721 mapped to both the sense and antisense strand of Naa40 (Figs 2B and S2A,B). Recently, additional functions of DNA have been identified. Exogenously introduced short (22 bp) and long (470 bp) dsDNA that were complementary to the tobacco mosaic virus RNA have been shown to inhibit RNA virus infection in plants^21^. Moreover, plants exposed to high concentrations of plant extracellular DNA derived from damaged cells exhibited growth inhibition owing to damage-associated molecular pattern-like signaling^31^. Based on these findings, we hypothesized that cgDNA functions as a gene expression regulator via recognition by unknown yet specific sensor proteins. To address this issue, we first treated cells with previously reported cgDNA and examined whether mRNA inhibition occurred. The experimental scheme is shown in Fig. 2C. cg721 was subcloned into pcDNA3 plasmid and extracted from the gel (Fig. S2C). Linearized dsDNAs were transfected into C2C12 cells (4 nM in 24-well plates). The RNA expression levels of 721 and Cox8a were not affected; however, Naa40 was inhibited (Fig. 2D–F), indicating that the transfected linearized 721 dsDNA (ds721) triggers DNA interference in the nucleus by targeting the Naa40 intron. This weak suppression by the transfected ds721 could be due to a combination of high melting temperature preventing the unwinding of dsDNA into ssDNA, and the existence of the mature mRNA prior to treatment (ds721 targets at the intron), or other reasons. To prevent the possibility that unremoved transfected ds721 affected the results presented in Fig. 2, serially diluted ds721 was treated with DNase, followed by cDNA synthesis and RT-qPCR analysis. The DNase-untreated dilutions demonstrated a one cycle decrease in Ct value per two-fold increase in concentration (Fig. S2D; left three lanes). There was no relationship among DNase-treated samples for Ct cycles (Fig. S2D; right three lanes) with a significant delayed fluorescence signal (Ct = 32.8 to 33.9). These results indicate that the DNase treatment used was sufficient to digest the added ds721. Thus, we concluded that our results were not due to some technical problem.

### cgDNA is a Natural Antisense to Regulate Cell Survival

**Figure 3:**
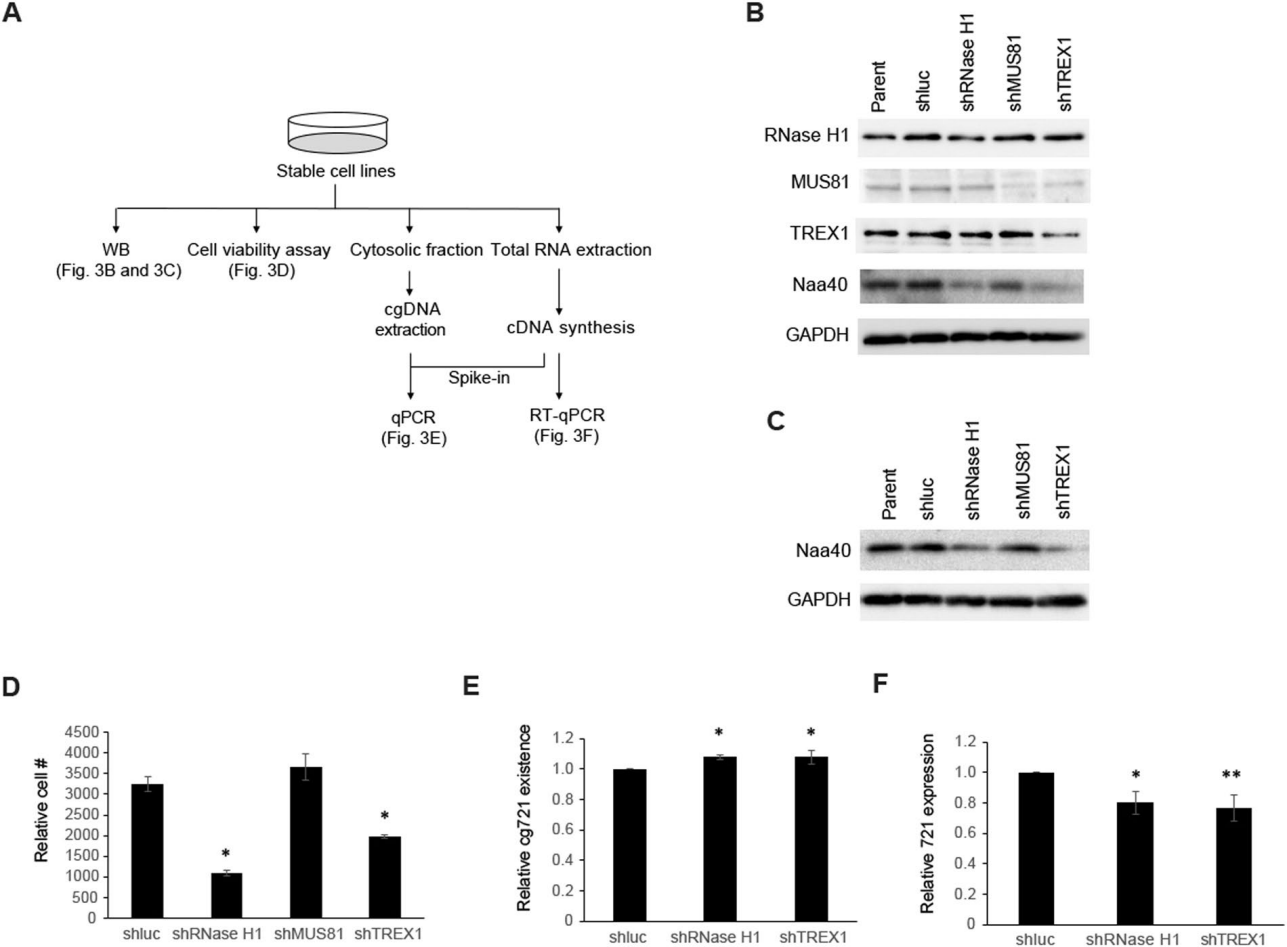
Inhibition of Naa40 reduces cell survival. (**A**) Schematic representation of experiments with shRNA cell. (**B** and **C**) Protein expressions of RNase H1, MUS81, and TREX1 in knocked down cells. (**B**) C2C12 cells were used to generate subcell lines stably expressing shRNA against *luciferase*, *RNase H1*, *MUS81*, and *TREX1*. Immunoblots performed with extracts from shRNA knocked down C2C12 cells. (**C**) Immunoblots performed with extracts from transient shRNA knocked down Hepa1-6 cells. (**D**) For proliferation studies, cells were plated and incubated overnight, and baseline counts were taken the following day (Day 0). Cell populations at Day 3 were normalized to those at Day 0. Cell counts for shluc versus shRNaseH1, shMUS81 and shTREX1 expressing C2C12 cells (**p < 0.01 by ANOVA/Bonferroni test; n = 3; mean ± SD). (**E**) Quantitative analysis (qPCR) of abundance of 721 in the cytosolic fractions (*p < 0.01 by ANOVA/Bonferroni test; n = 4; mean ± SD). (**F**) Quantitative analysis (RT-qPCR) of the 721 expression in knocked down cells (*p < 0.05 and **p < 0.01 by ANOVA/Bonferroni test; n = 4; mean ± SD).

To investigate whether cgDNA functions as a natural antisense, we established stable RNase H1, MUS81, and TREX1 knockdown mouse cell lines using short hairpin RNA (shRNA) in C2C12 cells. The experimental scheme is shown in Fig. 3A. We aimed to knockdown these three genes because (1) previously cloned cytosolic DNA contained retroelements and that could potentially form non-B DNA structures (R-loops). R-loops act as regulators of gene expression and are more susceptible to DNA damage, leading to DSB and recombination. RNase H1 reduces cellular R-loops and the absence of RNase H1 and its binding partner RPA enhances genomic instability^32,33^. (2) The structure-specific endonuclease MUS81 is involved in DSB in response to replication inhibition^34^. Genomic DNA cleavage by the MUS81 and PARP dependent DNA repair pathways leads to the accumulation of cgDNA in prostate cancer cells^12^. (3) TREX1 is known to digest cytosolic ssDNA to prevent autoimmune responses. TREX1 anchors within the outer nuclear membrane to ensure immediate degradation of ssDNA leaking into the cytosol^11,35,36^ and directly binds PARP1 during DNA damage^37^. The protein expression levels of Naa40 were mainly attenuated in the RNase H1 and TREX1 knocked down C2C12 cells (Fig. 3B). shRNase H1 cells failed to remove R-loops and shTREX1 cells were no longer able to degrade ssDNA. Thus, both shRNA expressing cells generated increased levels of cgDNA, which inhibited Naa40 expression. Naa40 up-regulation was not observed in shMUS81 cells. These results were reproduced even upon transiently knocked down in Hepa1-6 cells (Fig. 3C). To investigate whether cgDNA-dependent Naa40 suppression is associated with cell phenotype, we conducted cell viability assays with stable cells since Naa40 is known to be related to apoptosis in cancer cells^38,39^. Assay results demonstrated that while shRNase H1 and shTREX1 cells inhibited cell proliferation, shMUS81 cells did not (Fig. 3D), indicating that the lack of RNase H1 and TREX1 promoted increased cgDNA generation and reduced cell survival. On the other hand, MUS81 is related to DSB, and under the physiological conditions or in the absence of genotoxic reagents, MUS81 does not play a major role in ssDNA metabolism. Next, we wanted to determine the importance of RNase H1 and TREX1 in cellular cg721 metabolism; we performed quantitative PCR (qPCR) with the DNA extracted from cytosolic fractions. The cgDNA template was normalized to 30 ng per reaction as we previously executed in Fig. 1 and cDNA synthesized from total RNA was added to each sample as spike-in controls to minimize technical bias (Fig. 3A). The results analyzed by qPCR showed that the levels of cg721 in the cytosolic fractions were increased in shRNase H1 and shTREX1 knocked down C2C12 cells (Fig. 3E), whereas those from RT-qPCR showed that the expression levels of 721 transcripts were suppressed in the same cells (Fig. 3F). Taken together, these results indicated that cg721 is modulated by RNase H1 and TREX1 and the generated cg721 regulates not only Naa40 expression but also its own transcripts.

### Extracellular vesicles carry cgDNA to suppress the target gene expression in the proximal cells

**Figure 4:**
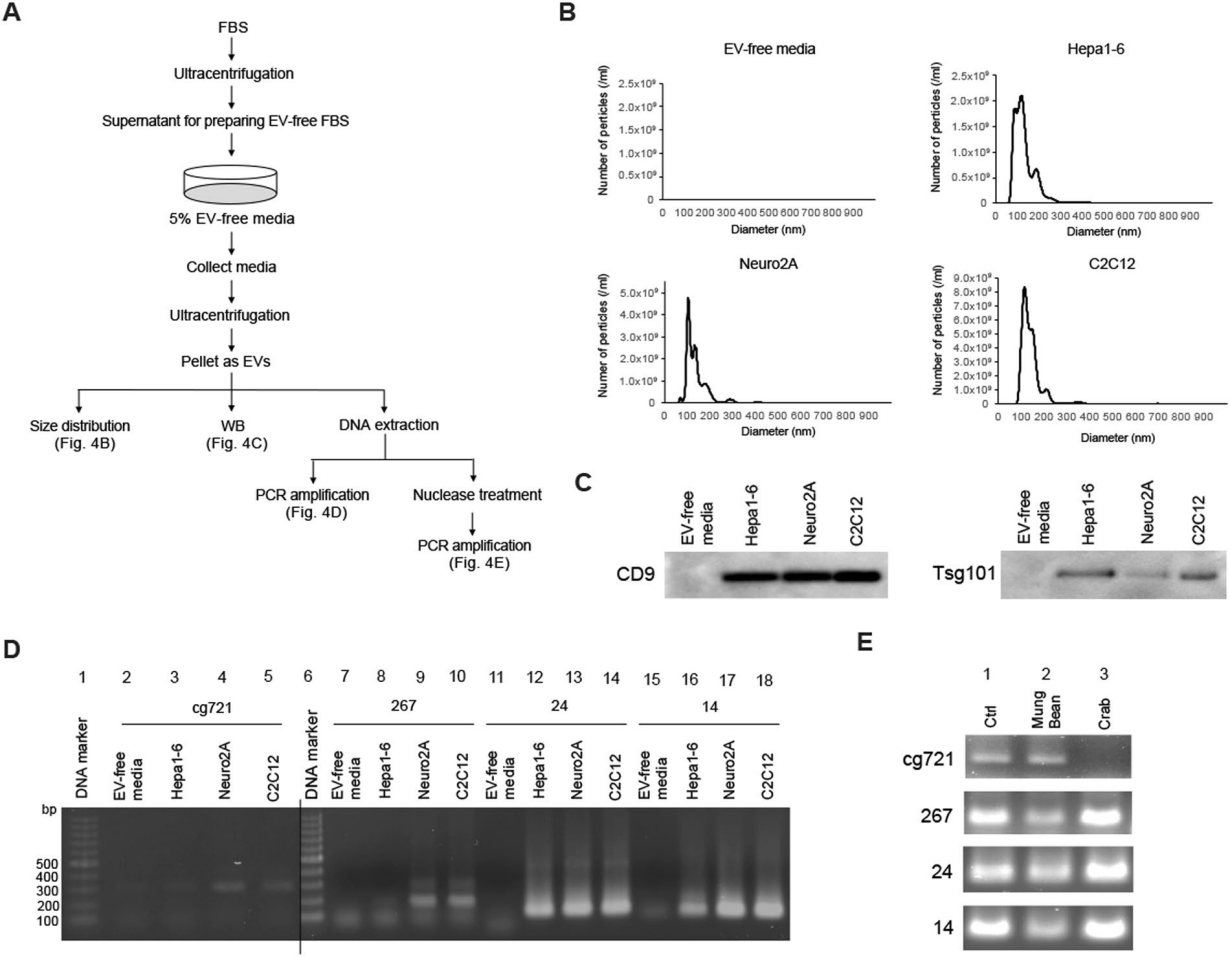
Cytosolic genomic DNA is incorporated in extracellular vesicles. (**A**) Schematic representation of the experiment. (**B**) Size distribution of extracellular vesicles in different cell lines. (**C**) Immunoblots performed with Hepa1-6, Neuro 2A, and C2C12 EVs. (**D**) PCR-amplification of DNA in extracellular vesicles. (**E**) Strand-specific Mung Bean and Crab nuclease treatments for DNA extracted from extracellular vesicles.

Cancer cells secrete more exosomes than normal cells, in correlation with tumor metastasis. Exosomes are taken up by the target cells to affect cell characteristics^40–42^. siRNA-related components, including pre-miRNA, miRNA, and the RISC complex as well as DNA are incorporated into exosomes^43^. Cytosolic DNA partially co-localizes with CD63, an exosome marker, which recycles to the cell via the exocytic pathway^7^. This raises the possibility of cgDNA secretion from exosomes. In addition, tumor exosomes carry genomic DNA and retrotransposon RNA transcripts^43–46^. Thus, we decided to collect extracellular vesicles (EVs) from Hepa1-6, Neuro2A, and C2C12 cells to investigate whether cgDNA was incorporated into the EVs. The experimental scheme is shown in Fig. 4A. Penicillin-streptomycin (PS) and trypsin-EDTA used for cell culture contained nanoparticles. To minimize the contamination from such reagents, we adapted and cultured cells in 5% EV-free FBS media and without PS. For each cell splitting, cells were washed by PBS three times to completely remove the residual trypsin-EDTA. The particle size of EVs collected from the EV-free media was determined by nanoparticle tracking analysis (NanoSight) as reported previously^47^. The prepared EV-free media had no detectable nanoparticles (Fig. 4B, top left). The media collected from Hepa1-6, Neuro2A, and C2C12 cells showed the nanoparticle size ranging from 30 to 800 nm, mainly around 100 to 200 nm (Fig. 4B, top right and bottom two), hence indicating that the secreted vesicles from the cells are EVs. Therefore, we aimed to detect EV makers by immunoblotting. Tetraspanins such as CD9, CD63, or Alix, Flotillin-1, and Tsg101 are known to be as exosome markers^48–50^. Collected EVs were immunoblotted against anti-CD9 and anti-Tsg101 antibodies. CD9 and Tsg101 were detected from the three cell lines, while EV-free media were not (Fig. 4C; left and right). Taken together, the collected EVs contained exosomes. For the further investigation on whether cgDNA is secreted via EVs, cells were treated with an nSMase2 inhibitor GW4869^51^ (Fig. S3A). EV-derived cg721 was amplified in the absence of GW4869, but not in its presence (Fig. S3B, lanes 2 and 3); however, the secretion of 267, 24, and 14 were only slightly inhibited by GW4869 (Fig. S3B, lanes 4 versus 5, 6 versus 7, 8 versus 9). This indicates that while cg721 is carried by EVs such as exosomes, 267, 24, and 14 may be carried by non-exosomal EVs in C2C12 cells. Interestingly, secreted EVs from Neuro2A and C2C12 cells contained all the tested cgDNAs (Fig. 4D), whereas those from Hepa1-6 cells carried transposon cgDNA 24 and 14, and very small amount of cg721 and 267 (Fig. 4D). EVs are considered as cell-specific and thus, classified based on their cellular origin as well as their packaging components and biological functions. To address whether the observed phenomenon was caused by the cell-type specific EVs secretion, collected EVs were immunocaptured by CD9 or Tsg101. The captured EVs were eluted from the bead, DNA extracted, and PCR-amplified (Fig. S3C). The expression levels of cg721 were low in Hepa1-6 and Neuro2A cells with CD9- and Tsg101-captured samples, except for Neuro2A flow-through (FT) of Tsg101-captured sample (Fig. S3D, top; lanes 1, 2, 3, 4, 8, 9, 10, 11). The protein expression levels of Tsg101 in Neuro2A was low compared to the other two cell lines (Fig. 4C), therefore, uncaptured FT showed more amplified cg721 products. Although the Tsg101 captured EVs showed higher cg721 expressions, we could detect cg721 in both CD9- and Tsg101-positive EVs in C2C12 cells (Fig. S3D, top; lanes 5, 6, 12, 13), indicating that these EVs carry cg721. As we shown in Figs 4D and S3D top, the expression levels of not only cg721 but also 267 were very low in Hepa1-6 cells (Fig. S3D, second from top; lanes 1, 2, 8, 9). On the other hand, 267 was abundant in CD9- and Tsg101-negative EVs in Neuro2A and C2C12 cells. This further demonstrated that 267 is not incorporated in exosomes since 267 was GW4869 insensitive (Fig. S3B). We did not observe significant differences in the expression of 24 and 14 among three cell lines. These cgDNA may or may not be incorporated into the exosomes (Fig. S3D, bottom two). Collectively, the mechanism of cgDNA secretion was shown to be cgDNA-origin specific as well as cell-dependent. To verify whether the secreted cgDNA was single- or double-stranded, the extracted DNA was treated with either a double-stranded specific Crab nuclease (Evrogen) or a single-stranded specific Mung Bean nuclease (Takara). Fig. S4A outlines the DNA oligos used to determine the specificity. As shown in Fig. S4B, Crab nuclease was able to digest completed, mismatched, and deletion mutant dsDNA, but failed to cleave ssDNA. Mung Bean nuclease cleaved ssDNA, but none of the dsDNA. The two different sequences gave identical results (Fig. S4B,C). Taken together, both nucleases used were strand-specific, but not structure-specific. DNA secreted from C2C12 cells was extracted from EVs, treated by nucleases, followed by PCR amplification (Fig. 4E). Amplification in Mung Bean nuclease-treated samples was similar to that in untreated samples (Fig. 4E upper panel, lanes 1 and 2). No amplification was apparent in the Crab nuclease-treated samples (Fig. 4C upper panel, lane 3), indicating that cg721 was incorporated into EVs in a double-stranded structure. Transposon cgDNA existed predominantly as ssDNA into the EVs (Fig. 4C lower three panels), consistent with previous studies, demonstrating that endogenous retroelements are sensitive to the single-stranded specific nuclease TREX1^11,35^.

**Figure 5:**
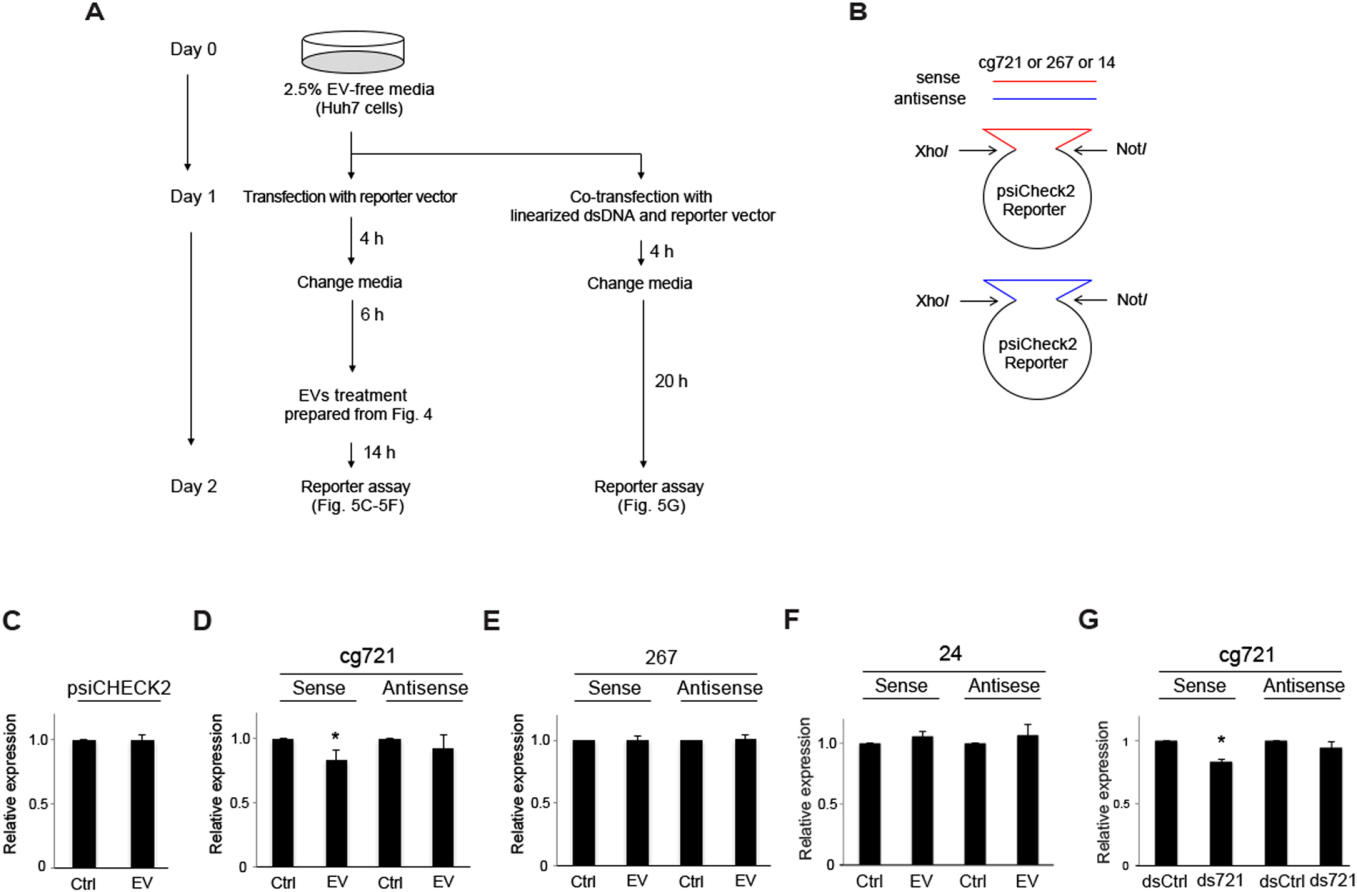
Exosomes-dependent target gene silencing. (**A**) The experimental scheme of silencing assays. (**B**) The design of reporter vectors. (**C**–**F**) (**C**) Relative expression of a psiCHECK2 empty luciferase reporter treated with EVs, (**D**) 721 subcloned reporter, (**E**) 267 subcloned reporter, (**F**) 24 subcloned reporter (p < 0.05; n = 4; mean ± SD). (**G**) Relative expression of the 721 subcloned luciferase reporter transfected with dsDNA (p < 0.05; n = 5; mean ± SD).

**Figure 6:**
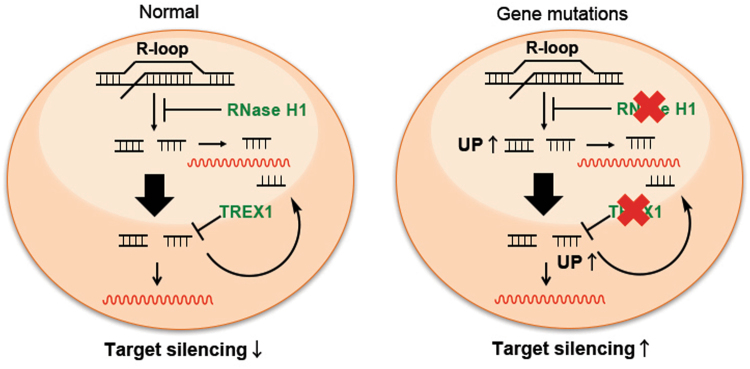
A model for cgDNA-dependent gene silencing.

To determine whether EV-derived cgDNA is taken up by cells to silence the target gene expression, we conducted luciferase reporter assays using sense and antisense cg721, transposon cgDNA 267, and 14. The experimental designs are shown in Figs 5A,B and 6. It is possible that the secreted EVs are taken up by the cells and inhibit reporter. To prevent this technical self-inhibition, in the precise evaluation of whether secreted EVs act as a transmitter to silence the target transcripts of proximal acceptor cells, we chose human hepatocellular carcinoma Huh7 cells as a reporter plasmid-expressing cells. The human genome aligned with none of the mouse cgDNA, indicating that secreted human EVs from Huh7 cells do not silence the transfected mouse cgDNA reporter in our assay. The expression levels of Renilla luciferase, without target sites, were not affected by the exogenously added EVs prepared from EV-free media (Ctrl) and from C2C12 cells (EV), and endogenously secreted human exosomes from Huh7 (Fig. 5C). This suggested that the reporter assays we established were sensitive to reporter sequences. Cells expressing the cg721 sense strand suppressed the expression of the luciferase reporter (Fig. 5D, left); however, expression of the antisense strand did not result in silencing (Fig. 5D, right). Luciferase expression was not attenuated by the transposon elements (Fig. 5E and F). To further confirm if silencing is a strand-specific, reporter assays with agarose gel purified ds721 were conducted. Cells expressing the sense strand, but not the antisense strand of the reporter gene, silenced luciferase expression (Fig. 5G). These results suggested that dsDNA-dependent gene regulation is a strand-specific via unknown mechanisms.

## Discussion

Pathogenic, genotoxic reagent sensitive and tumor-derived cgDNA induces inflammatory signaling pathways. The DNA/RNA sensing pathway is integrated by an adaptor protein STING, followed by the induction of immune responses^3,52,53^. The DNA sensing pathway is critical for not only the immune system, but also for senescence and senescence-associated secretory phenotypic factors are regulated by cGAS-STING^54,55^. Cellular senescence is an anti-proliferative program that is triggered by telomere shortening, DNA damage, oncogene activation, and even cancer^56,57^. The balance of cGAS-STING pathway activation and TREX1 activation controls cytosolic dsDNA accumulation triggered by a radiation^6^. A model for cgDNA-dependent gene silencing is shown in Fig. 6. In our study, both RNase H1 and TREX1 knocked down cells, with increased cgDNA abundance, showed slow proliferation by inhibiting Naa40, since the latter is known to be associated with apoptosis in cancer cells^38,39^. A key finding of the study is that cgDNA was detected in three mouse cell lines under physiological conditions, and cgDNAs were incorporated into EVs. cg721 suppressed the target gene expression in the cells as well as in the proximal cells via EVs with a strand selection and the inhibiter experiments with nSMase2 showed that there were different mechanisms of cgDNA secretion. Cell-free DNA (cfDNA) is one of the blood biomarkers, especially used for patients with cancer, to predict metastasis, treatment response, recurrence, prognosis, and diagnosis^58,59^. Tumor cells secrete more exosomes than normal cells, and DNA repeat sequences such as transposons are released by tumor cells^60^. Systemic lupus erythematosus (SLE) is an autoimmune disorder. In patients with SLE, induction of autoimmunity is initiated by immune recognition of endogenous nucleic acids such as DNA and histones. Endogenous retroelements represents novel paradigms of SLE^61^. TREX1 prevents the L1 retrotransposon-induced DNA damage response, and mutations in RNases and a DNase, TREX1 has been linked with SLE, also known as Aicardi-Goutières syndrome^62–65^, and other autoimmune diseases such as Sjögren’s syndrome and systemic sclerosis^65^. R-loops are three-stranded DNA/RNA structure that act in gene expression regulation and induce genome instability leading to DSB. DNA/RNA helicases and RNase H remove the generated R-loops^66^. TREX1 localizes on the outside of the nucleus and is recruited to the nucleus in response to DNA damage and apoptosis^35,37,67^. RNase H1 and TREX1 are known to metabolize cgDNA. Once this mechanism is disrupted, cgDNA accumulates in the cytosol and may induce apoptosis or senescence, and an inflammatory response. Although, till date, cg721 has not been annotated as a transposon element, its sequence mapped to both Naa40 sense and antisense strands, suggesting that it could be a transposon. More than half of the human genome consists of transposable elements^68^. They play an important role in DNA repair, gene regulation, genome evolution, epigenetics, histone methylation, and diseases^69–76^. cg721 plays multiple roles in the regulation of gene silencing as a natural antisense and in cell-to-cell gene regulation, and cgDNAs may also constitute important key components of autoimmune diseases once it secreted out of the cell, where they function as immune response antigens.

## Methods

### Fractionation and cytosolic DNA and RNA extraction

Cells cultured with DMEM containing 10% FBS were trypsinized and centrifuged to produce a pellet. Four volumes of lysis buffer (20 mM HEPES, pH 7.4, 10 mM KCl, 2 mM MgCl_2_, 1 mM DTT, and 1 mM EDTA) were added to the pellet and incubated for 20 min on ice. Cells were passed 15 times through a 25 gauge needle to enhance cell lysis. The pellet suspension was centrifuged twice at 800xg for 5 min each. The pellet fraction was resuspended in a lysis buffer and used as the nuclear fraction. The supernatant was centrifuged twice at 10,000xg for 10 min. The pellet fraction was resuspended in a lysis buffer and used as the mitochondria fraction. The collected supernatant was used as the cytoplasmic fraction. The nuclear, mitochondrial, and cytoplasmic fractions were incubated in lysis buffer for 1 h at 37 °C for the further extraction, as previously reported^7^ with minor modifications (50 mM HEPES, pH 8.0, 150 mM NaCl, 10% glycerol, 1 mM DTT, 2 mM EDTA, 0.5% SDS, and 100 μg/mL proteinase K (Sigma-Aldrich)). DNA was extracted with phenol:chloroform:isoamyl alcohol (PCI) (Nippon Gene) and RNA was extracted with acid phenol:chloroform (Thermo Fisher Scientific). For intron PCR, RNA was treated with DNase I (Wako Pure Chemical Industries) for 1 h at 37 °C followed by acid phenol:chloroform extraction. For PCR of cytosolic genomic DNA PCR, DNA was treated with an RNase cocktail (Thermo Fisher Scientific) for 1 h at 37 °C followed by PCI extraction. All purified DNA were adjusted to a concentration of 6 ng/μl for the PCR amplification. The sequences of the primers used are shown in Table S2.

### Sequences of cytosolic genomic DNA

cg721 sequence was previously reported by Shen *et al*., as shown in the Fig. 4F of their report^7^. We referred to the bold letter region (286 bp in length) as cg721 that was predicted as a putative triplex form. The sequences of transposon DNA 267 (*MaLR*, *ORRA1*, and antisense to the *Ptprm* intron), 24 (L1), and 14 (antisense to *MERV-L*) were previously cloned by Stetson *et al*. and the detailed cloning method is depicted in Fig. 5, and the related sequences of transposon DNAs are presented in supplemental data, Table S1 of their report^11^.

### Double-stranded DNA preparation and cell treatment

pBR322 vector was digested with Hind*III* and BamH*I* restriction enzymes to obtain 346 bp double-stranded DNA. cg721 was subcloned into pcDNA 3 vector between Hind*III* and EcoR*I* sites. The correct clone was digested and the fragments were detected on an agarose gel visualized using ChemiDoc Touch Imaging System (Bio-Rad). The correct bands (346 bp for Ctrl dsDNA and 296 bp for cg721; 10 bp longer than the original cg721 because of the additive restriction sites) were cut out with a razor blade and DNA was extracted from the gel. Cells cultured in 24-well plates were transfected with lipofectamine 2000 and linearized dsDNA at a final concentration of 4 nM. All restriction enzymes were purchased from New England BioLabs (NEB).

### Quantitative real-time PCR assay

Total RNA was extracted from cultured cells using QIAzol (Qiagen). Extracted RNA was treated with DNase I followed by acid phnenol:chloroform extraction. RNA was reverse transcribed with Transcriptor Universal cDNA Master (Roche). Quantitative real-time PCR assay was performed with as previously described^22^. PCR primers and probes to detect the expression levels of Cox8a and Naa40 were purchased from Applied Biosystems and primers and probe for the 721 were shown in Table S2.

### shRNA and stable cell lines

DNA oligos for shRNA were cloned into pBAsi-mU6 Pur vector (Takara). The shluc shRNA has been previously characterized^19^. The other shRNA sequences for each gene were chosen from validated sequence from Mission shRNA (Sigma-Aldrich) sequences. All sequences used are detailed in Table S2.

### Cell proliferation assays

Cells were plated and incubated overnight. The CellTiter-Glo luminescent cell viability assay (Promega) was conducted to measure cell numbers as previously reported^19^. Baseline counts were taken on the next day (day 1). Cell populations at day 3 were normalized to those at day 1.

### Quantitative PCR assay

Cytosolic genomic DNA from shRNA knocked down C2C12 stable cells were prepared as previously described in the “Fractionation and cytosolic DNA and RNA extraction” section. Spike-in cDNA was prepared as previously described in the “Quantitative real-time PCR assay” section to minimize the qPCR technical bias and for normalization. Prepared both DNA were mixed in a single tube and amplified by specific primers to measure the abundance of cytosolic genomic DNA. Primers used for the assay were shown in Table S2.

### Extracellular vesicle-free FBS preparation

Fetal bovine serum (FBS) was centrifuged at 35,000 rpm for overnight (>16 h) (Beckman SW 41 Ti swing-bucket rotor) to remove bovine-derived exosomes. The supernatant was filtered using a 0.22 μm filter. Extracellular vesicle-free FBS (EV-free FBS) was added to DMEM to make a final concentration of either 2.5% or 5% EV-free FBS DMEM without penicillin-streptomycin.

### Extracellular vesicle preparation, purification, characterization and analysis

On the day prior to the experiment, cells were split with 5% FBS DMEM to achieve 80% confluence the next day. Cells were washed three times with PBS prior to the experiment to remove all the residual components and replaced with 5% EV-free FBS DMEM. 24 h post-replacement, media were collected and centrifuged at 3,000 rpm for 5 min to remove any possible apoptotic bodies and cell debris. The supernatants were filtered by using a 0.22 μm filter and centrifuged at 35,000 rpm for 70 min. The pellet was washed with sufficient PBS and centrifuged at 35,000 rpm for 70 min to re-pellet. The prepared EVs were analyzed by using a nanoparticle characterization system (NanoSight) as previously described^47^, and western blotting with anti-CD9 (ab927726, Abcam) and Tsg101 (GTX118736, Gene Tex) antibodies. “Control EVs” were prepared following the same protocol from EV-free FBS media and used as controls in the experiments. Secreted cgDNA was extracted by PCI and treated with RNase cocktail to remove RNA.

### Inhibition of exosomes secretion by nSMase2 inhibitor GW4869

C2C12 cells were cultured in 5% EV-free media. Cells were treated with GW4869 (final 3 μM) for 48 h and media were collected. The collected media were processed as described above in “Extracellular vesicles preparation, purification, characterization and analysis” section. Extracted EV DNA was amplified by PCR using specific PCR primers.

### Extracellular vesicle immunoprecipitation

EVs were suspended in PBS and incubated with either CD9 or Tsg101 antibody for overnight. Uncaptured EVs were collected as Flow-through (FT) samples and the captured vesicles were washed with PBS for 5 times. EVs were eluted by a 300 μL of 0.2 M glycine (pH 2.0) for 10 min and immediately neutralized by adding a 150 μL of 1 M Tris-HCl (pH 8.0). DNA was extracted from both FT and neutralized eluents by PCI. The extracted DNA was treated with an RNase cocktail and amplified using specific PCR primers.

### ssDNA and dsDNA specific nuclease treatment

EVs DNA extracted by PCI and treated with RNase cocktail was treated with either ssDNA specific Mung Bean Nuclease (Takara) or dsDNA specific Crab Nuclease (Evrogen) for 1 h at 37 °C followed by PCI extraction. The treated DNA was amplified by PCR with specific primers to determine ssDNA or dsDNA and resolved on agarose gels.

### Silencing assays

Silencing assays were conducted by co-transfecting human liver carcinoma cell line Huh7 with constructs encoding either wild-type Renilla luciferase or Renilla luciferase under the control of the full length of cgDNA (cg721, 267, or 14) target site encoded at the 3′-UTR of psiCHECK2 vector (Promega). Firefly luciferase was used as a transfection control. Reporter activity was assayed by using a Dual Luciferase Reporter System (Promega). Huh7 cells were adapted and cultured in 2.5% EV-free media and without penicillin-streptomycin (PS). A day before the experiment, cells were trypsinized and washed by PBS three times to get rid of any EVs coming from trypsin-EDTA (Nakarai) and split with 2.5% EV-free media and without PS. For the EV-mediated silencing assays, the cells were transfected with the reporter vectors using lipofectamine 2000 and the media were replaced at 4 h post-transfection by the EV-free media. After 6 h, prepared EVs from the section of “Extracellular vesicles preparation, purification, characterization and analysis” were added to each transfected cell and the cells were harvested at 14 h post-treatment to analyzed the expression levels of luciferase. For the dsDNA-mediated silencing assays, cells were co-transfected with the reporter vectors and linearized dsDNA (4 nM in 24-well plates) using lipofectamine 2000. The media were replaced at 4 h post-transfection and the cells were harvested and the expression levels of luciferase were analyzed at 24 h.

### Statistical analysis

Experiments were typically run in triplicate and repeated at least three times. Image quantitation was performed by using ImageJ analysis software (NIH). Data are presented as mean ± SD, unless otherwise stated. Student’s t tests were employed where the minimum level of significance was p < 0.05.

### Data availability

The datasets analyzed in the current study are available from the corresponding author upon reasonable request.

## Electronic supplementary material

Dataset 1
