# Supplementary material for: Cytosolic Genomic DNA functions as a Natural Antisense
Source: Sci Rep. 2018 Jun 4;8:8551. doi: 10.1038/s41598-018-26487-1 (PMC5986806; doi:10.1038/s41598-018-26487-1)
Supplement: Supplementary file 1 — Dataset 1 [file 41598_2018_26487_MOESM1_ESM.docx]

**Title: Cytosolic Genomic DNA functions as a Natural Antisense**

**Authors:**

Ken Asada^1,2^, Keiya Ito^3^, Daishi Yui^1,2^, Hirokuni Tagaya^3^, Takanori Yokota^1,2*^

**Affiliations:**

^1^Department of Neurology and Neurological Sciences, Graduate School of Medical and Dental Sciences, Tokyo Medical and Dental University, Tokyo, 113-8519, Japan.

^2^Center for Brain Integration Research, Tokyo Medical and Dental University, Tokyo, 113-8519, Japan.

^3^Department of Health Science, School of Allied Health Sciences, Kitasato University, Kanagawa, 252-0373, Japan.

*Correspondence: tak-yokota.nuro@tmd.ac.jp (T.Y.)

**Supplemental Information:**

**Figure S1.**


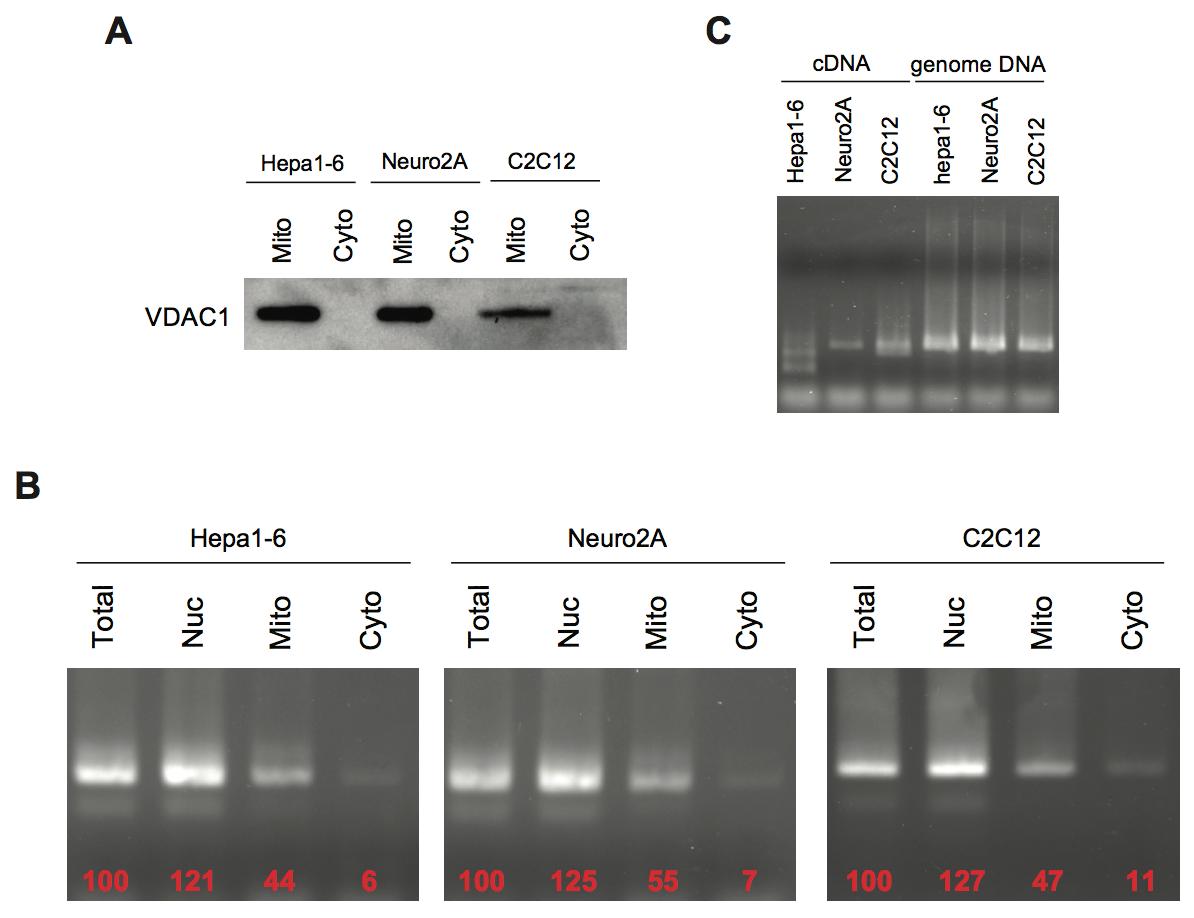


**Figure S1.** The cytosolic fraction does not contain mitochondria components.

(A) Immunoblots of mitochondria and cytosolic fractions. (B) 721 sequences in total, nuclear, mitochondria and cytosol fractions from Hepa1-6, Neuro2A, and C2C12 were amplified by PCR. The numbers at the bottom of the image indicate the signal intensity analyzed by ImageJ normalized to the total. (C) Total RNA were extracted followed by the treatment of DNase. Extracted RNA were reverse-transcribed into cDNA. Synthesized cDNA was used for PCR amplification to amplify the genome region of 7,218,966 to 7,219,251. Genome DNA was used for PCR control.

**Figure S2.**

**Figure S2.** 721 sequence is aligned to Naa40 using NCBI BLAST.

(A and B) Alignments of the sense strand of 721 sequence with (A) the Naa40 sense strand and (B) the Naa40 antisense strand. (C) Preparation of dsDNA preparation for DNA interference studies. (D) 721 dose-dependent Ct values with or without of DNase treatment.

**Figure S3.**

**
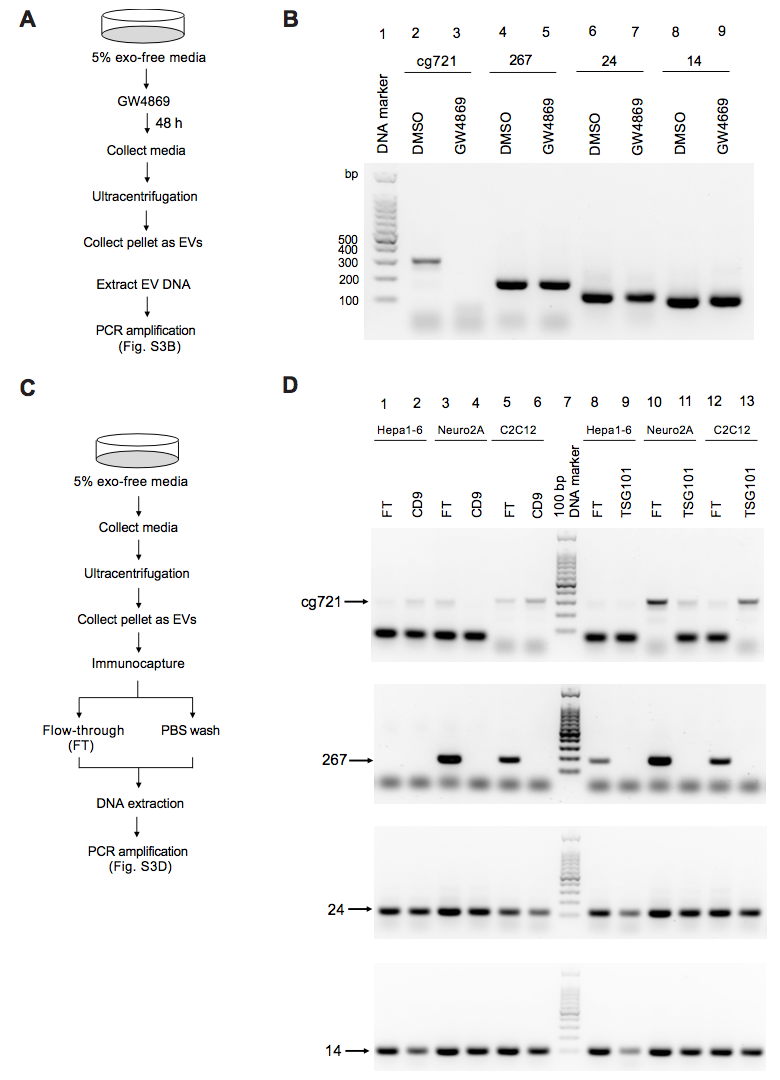
**

**Figure S3.**

Mechanism of secretion of cgDNA are cgNDA-origin specific and cell-dependent.

(A) The experimental scheme of extracellular vesicles secretion inhibition by GW4869. (B) Amplification of EV DNAs by PCR. (C) The experimental scheme of immunocapture of EVs. (D) Amplification of captured EVs by PCR.

**Figure S4.**

**Figure S4.** Strand specific nuclease DNA degradation.

(A) Oligos designed for the experiments. (B and C) (B) Double-stranded specific Crab nuclease and (C) single-stranded specific Mung Bean nuclease were used to treat the oligos. The sequences used are detailed in Table S1.

**Table S1: Known cytosolic DNA sensor proteins, related to** **Figure 6.**

| No. | Identified protein | Year | Title |
| --- | --- | --- | --- |
| 1 | TLR9 | 2000 | A Toll-like receptor recognizes bacterial DNA. |
| 2 | DLM-1/ZBP1 | 2007 | DAI (DLM-1/ZBP1) is a cytosolic DNA sensor and an activator of innate immune response. |
| 3 | AIM2 | 2009 | An orthogonal proteomic-genomic screen identifies AIM2 as a cytoplasmic DNA sensor for the inflammasome. |
| 4 | RNA Pol-III/RIG-I | 2009 | RNA Polymerase III Detects Cytosolic DNA and Induces Type I Interferons through the RIG-I Pathway.  RIG-I-dependent sensing of poly(dA:dT) through the induction of an RNA polymerase III-transcribed RNA intermediate. |
| 5 | LRRFIP1 | 2010 | The cytosolic nucleic acid sensor LRRFIP1 mediates the production of type I interferon via a β-catenin-dependent pathway. |
| 6 | DHX9/DHX36 | 2010 | Aspartate-glutamate-alanine-histidine box motif (DEAH)/RNA helicase A helicases sense microbial DNA in human plasmacytoid dendritic cells. |
| 7 | Ku70 | 2011 | Ku70 Is a Novel Cytosolic DNA Sensor That Induces Type III Rather Than Type I IFN. |
| 8 | DDX41 | 2011 | The helicase DDX41 senses intracellular DNA mediated by the adaptor STING in dendritic cells. |
| 9 | STING | 2011 | STING is a direct innate immune sensor of cyclic di-GMP. |
| 10 | DNA-PK | 2012 | DNA-PK is a DNA sensor for IRF-3-dependent innate immunity. |
| 11 | LSm14A | 2012 | LSm14A is a processing body-associated sensor of viral nucleic acids that initiates cellular antiviral response in the early phase of viral infection. |
| 12 | cGAS | 2013 | Cyclic GMP-AMP Synthase Is a Cytosolic DNA Sensor That Activates the Type I Interferon Pathway. |
| 13 | MRE11 | 2013 | DNA damage sensor MRE11 recognizes cytosolic double-stranded DNA and induces type I interferon by regulating STING trafficking. |
| 14 | IFI16 | 2014 | IFI16 DNA Sensor Is Required for Death of Lymphoid CD4 T Cells Abortively Infected with HIV. |
| 15 | Rad50-CARD9 | 2014 | Rad50-CARD9 interactions link cytosolic DNA sensing to IL-1b production. |
| 16 | DHX29 | 2014 | Helicase proteins DHX29 and RIG-I cosense cytosolic nucleic acids in the human airway system. |
| 17 | Sox2 | 2015 | Sox2 functions as a sequence-specific DNA sensor in neutrophils to initiate innate immunity against microbial infection. |
| 18 | BAF | 2015 | BAF is a cytosolic DNA sensor that leads to exogenous DNA avoiding autophagy. |
| 19 | DDX60 | 2016 | Cytosolic DNA Sensor Upregulation Accompanies DNA Electrotransfer in B16.F10 Melanoma Cells. |

**Table S2: Oligos used to analyze cgDNA, RT-qPCR, generate constructs for reporter vectors, shRNA vectors, and to investigate the strand specific nucleases, related to** **Figure 1 - Figure 5 and Figure S1 - S4.**

| **Primers for cgDNA** | **Primers used to detect cgDNA** |
| --- | --- |
| GAPDH intron forward primer | 5′ gccaaagacagaagccaggag 3′ |
| GAPDH intron reverse primer | 5′ actgcatcatcgaacctctcc 3′ |
| 721 forward primer | 5′ tttaagcttttctcaatatcgcttgttttttgt 3′ |
| 721 reverse primer | 5′ tttgaattcgaagatttaccaggctggcctcaa 3′ |
| Transposon 267 forward primer | 5′ tttaagcttggatagacagacattgtgatgatt 3′ |
| Transposon 267 reverse primer | 5′ tttgaattcagttctgcatcttcatctgaaggc 3′ |
| Transposon 24 forward primer | 5′ tttaagcttccgcctcacgagccaggcggacac 3′ |
| Transposon 24 reverse primer | 5′ tttgaattcagaagcacagagccgctgaggcag 3′ |
| Transposon 14 forward primer | 5′ tttaagcttccaagtgagggatgtgttgtctcc 3′ |
| Transposon 14 reverse primer | 5′ tttgaattcctggcccctcccacaaccaagaaa 3′ |
| **Primers for qPCR** | **Primers used to analyze cg721 existence by qPCR** |
| 721 forward primer | 5′ tttaagcttttctcaatatcgcttgttttttgt 3′ |
| 721 reverse primer | 5′ tttgaattcgaagatttaccaggctggcctcaa 3′ |
| **Primers for RT-qPCR** | **Primers used to analyze 721 expression levels by RT-qPCR** |
| 721 forward primer | 5′ gagatggctcagtgggtaagag 3′ |
| 721 reverse primer | 5′ aaactcagacactccagaagagg 3′ |
| 721 TaqMan probe | 5′ agttcaaatcccagcaaccacgtggtggct 3′ |
| **Primers for shRNA cell lines** | **Primers used to generate shRNA cell lines** |
| shluciferase forward primer | 5′ gatccgattatgtccggttatgtattcaagagatacataaccggacataatctttttta 3′ |
| shluciferase reverse primer | 5′ agcttaaaaaagattatgtccggttatgtatctcttgaatacataaccggacataatcg 3′ |
| shRNaseH1 forward primer | 5′ gatccgtagtgagcaaggacgcatttttcaagagaaaatgcgtccttgctcactactttttta 3′ |
| shRNaseH1reverse primer | 5′ agcttaaaaaagtagtgagcaaggacgcattttctcttgaaaaatgcgtccttgctcactacg 3′ |
| shMUS81 forward primer | 5′ gatccgtagtgcctggaagttcgaaacttcaagagagtttcgaacttccaggcactactttttta 3′ |
| shMUS81 reverse primer | 5′ agcttaaaaaagtagtgcctggaagttcgaaactctcttgaagtttcgaacttccaggcactacg 3′ |
| shTREX1forward primer | 5′ gatccacaaccaacctaaggccacatttcaagagaatgtggccttaggttggttgttttttta 3′ |
| shTREX1reverse primer | 5′ agcttaaaaaaacaaccaacctaaggccacattctcttgaaatgtggccttaggttggttgtg 3′ |
| **Primers for silencing assays** | **Primers used to generate reporter vectors for silencing assays** |
| 721 forward primer | 5′ tttctcgagttctcaatatcgcttgttttttgt 3′ |
| 721 reverse primer | 5′ tttgcggccgcgaagatttaccaggctggcctcaa 3′ |
| Transposon 267 forward primer | 5′ tttctcgagggatagacagacattgtgatgatt 3′ |
| Transposon 267 reverse primer | 5′ tttgcggccgcagttctgcatcttcatctgaaggc 3′ |
| Transposon 24 forward primer | 5′ tttctcgagccgcctcacgagccaggcggacac 3′ |
| Transposon 24 reverse primer | 5′ tttgcggccgcagaagcacagagccgctgaggcag 3′ |
| **Oligos for strand-specific nucleases** | **Oligos used to investigate for strand-specific nucleases** |
| 27mer - a: Sense | 5′ tttcatatgagggaatctgcagcacag 3′ |
| 27mer - a: Antisense | 5′ ctgtgctgcagattccctcatatgaaa 3′ |
| 27mer - a: Mismatch antisense | 5′ ctgtgctgcagaatccctcatatgaaa 3′ |
| 27mer - a: Deletion mutant antisense | 5′ ctgtgctgcagattccctcatatg 3′ |
| 27mer - b: Sense | 5′ tttgagctcctacacagtagcatcaaa 3′ |
| 27mer - b: Antisense | 5′ tttgatgctactgtgtaggagctcaaa 3′ |
| 27mer - b: Mismatch antisense | 5′ tttgatgctactgagtaggagctcaaa 3′ |
| 27mer - b: Deletion mutant antisense | 5′ gatgctactgtgtaggagctcaaa 3′ |
